# Work for the V.A.D.s.

**Published:** 1919-08-02

**Authors:** 


### PANEL DOCTORS AND THE HOSPITALS.

### Work for the V.A.D.s.

In the course of an animated discussion at the annual representative meeting in London of the British Medical Association (July 24-25), it was agreed that insurance practitioners were crippled in their work by the limitations of the present service, and that these limitations were also detrimental to its prestige. It was resolved that it was necessary to extend the scope of the insurance medical service to include (*a*) consultant or specialist non-residential services, (*b*) laboratories, (*c*) a nursing service. It was further agreed, on the motion of Dr. E. K. Le Fleming (Bournemouth), that in any scheme of medical reconstruction arrangements should be made for auxiliary hospitals in which general practitioners could treat their own patients.

In the course of his speech, Dr. Le Fleming said the principle behind the Bournemouth resolution was based on an article by Dr. Bottomley, of Bournemouth. They had in view, in the first place, the overcrowding of the recognised hospitals; and, in the second place, the fact that when an insured patient reached a certain stage of illness he passed out of the hands of the panel practitioner and went into the hospital. What they wanted was an auxiliary type of hospital to deal with cases that the panel doctor could not treat in their own homes; cases, for example, that did not require arduous nursing, but which had to be moved. Why should the V.A.D. service be entirely lost ? Surely there could be created auxiliary hospitals on V.A.D. lines, so that panel doctors might follow up their patients ?

In supporting the motion, Major Wallace Henry (Leicester) said a large number of practitioners took an interest in their patients after the latter entered the general infirmary. It was advisable to have beds available for such cases, if only to show how wrong was the idea that the panel practitioners sent the patients on to the hospital to get rid of them.

